# Prevalence and detection of drug resistant mutations in *Mycobacterium tuberculosis* among drug naïve patients in Nairobi, Kenya

**DOI:** 10.1186/s12879-019-3911-9

**Published:** 2019-03-25

**Authors:** Collins Otieno Ogari, Anthony Kebira Nyamache, James Nonoh, Evans Amukoye

**Affiliations:** 1 Microbiology Department, Kenyatta University, Nairobi, Kenya; 2 Kenya Medical Research Institute, Centre for Respiratory Disease and Research, Nairobi, Kenya

**Keywords:** Tuberculosis, Drug-resistance, Mutation, Kenya

## Abstract

**Background:**

Tuberculosis (TB), an ancient scourge of humanity known for several thousands of years, is still a significant public health challenge in many countries today even though some progress has been made in recent years in controlling the disease. The study’s aim was to determine the prevalence of mutations responsible for drug resistance in *Mycobacterium tuberculosis* among patients visiting selected health centers in Nairobi, Kenya.

**Methods:**

The cross-sectional study involved 132 TB positive patients visiting Mbagathi and Chandaria hospitals between September 2015 and August 2016. Sputum samples were collected from the participants and handled in a biosafety level 3 laboratory at the Kenya Medical Research Institute (KEMRI). Samples were decontaminated using N-Acetyl-L-Cysteine (NALC) – Sodium Hydroxide (NALC-NaOH), stained using Zeihl–Neelsen (ZN), and cultured in Mycobacterium Growth Indicator Tube (MGIT). DNA extracted from cultured isolates using Genolyse™ technique was subjected to Multiplex PCR amplification and reverse hybridization for detection of drug resistance mutations on *rpoB*, *katG*, *inhA*, *gyrA*, *gyrB*, *rrs* and *eis* genes using Hain Genotype MTBDR*plus* and MTBDR*sl*.

**Results:**

All 132 (100%) patients included in the study were culture positive for *M. tuberculosis*. Among them, 72 (54%) were male while the remaining 60 (46%) were female. The mean age of the patients was 26.4 ± 19.4 (SD) with a range of 18 to 60 years. Overall, the prevalence of the resistance to first and second-line TB drugs was 1.5% (2/132). Resistance to isoniazid (INH) was observed in 1 of 132 patients (0.8%), as was multi-drug resistant tuberculosis (MDR-TB), also at 0.8%. No resistance to fluoroquinolones (FQ) or kanamycin (KAN) was observed. The INH resistant strain had the *katG* mutations S315 T, while mutations detected for the MDR-TB were *katG* S513 T for INH, *rpoB* S531 L for rifampicin (RIF) and *rrs* G1484 T for cross-resistance to aminoglycosides/capreomycin (AG/CP).

**Conclusions:**

Molecular analysis confirms transmission of the drug-resistant *M. tuberculosis* strains. The data suggested that there is homogeneity when it comes to the type of drug resistance and mutation that occurs in the region. This calls for intensified drug resistance surveillance and drug adherence among patients infected with TB.

## Background

Despite global tuberculosis (TB) control interventions, TB is still a public health issue. TB infection is currently ranked among the top three killer infectious diseases worldwide [1]. The current situation on TB infection and drug resistance is due to failure in prompt diagnosis, poor access to resources and framework, poor drug adherence, suboptimal treatment, mismanagement of TB control programs, migration, poverty, increased population, and increased cases of TB/Human Immunodeficiency Virus (HIV) cross-infection [2].

Drug-resistant and multi-drug resistant (MDR) TB is characterized by mortality, inadequate treatment regimens, and delayed diagnosis. Implementations of strategies such as the Directly Observed Treatment Short Course (DOTS) is essential for both diagnosis and patient treatment, to avoid creation and spread of the resistant strains in the community [3]. However, some of the present strategies have limitations. For instance, sputum smear microscopy is useful to detect the primary sources of infection but only helps diagnosis of about half of all TB cases, culture is more sensitive and takes a longer time to produce results, and 6 to 8 months is still a long time to maintain a patient on regular treatment [4].

To understand the dynamics in transmission and to formulate novel anti-TB drugs, there is a need to explore global distributions of drug resistance mutations. This can inform a basis for understanding drug-resistant gene migrations within populations since the frequency of mutations vary geographically. Currently, there is limited data available on the patterns of *Mycobacterium* resistance gene mutation in Kenya. The survey of TB prevalence was first carried out in Kenya between 1958 to 1959. The second survey was carried out using GeneXpert technology from July 2015 to 2016 by the Ministry of Health. It was also interesting to note that the findings highlighted that about 40% of TB cases go unnoticed and untreated [5]. Since one infected and an untreated individual can end up spreading the infection to 10–15 individuals, these missing TB cases continue to fuel the spread of TB [5]. In 2018, the World Health Organization (WHO) estimated 319 TB cases per 100,000 people in Kenya [6].

Knowledge of the prevalence of geographic-specific mutations can allow the development of in-house, polymerase chain reaction (PCR) based methods for targeting mutations relevant in a specific setting. Besides, the drug-resistance data is also helpful in advising the process of developing new molecular assays for the diagnosis of MDR tuberculosis and formulation of novel drugs, a great concern, when considering the increasing rates of MDR and extremely drug-resistant (XDR) TB worldwide.

The purpose of the present study was to determine the prevalence and detection of drug resistant mutations in *M. tuberculosis* among patients visiting selected health centers in Nairobi, Kenya.

## Methods

### Study design and sample collection

A cross-sectional study was conducted among suspected TB patients visiting TB clinics of Mbagathi district, and Chandaria community hospitals. Stratified systematic sampling was carried out for the two sampled hospitals during the period between September 2015 and August 2016. Sputum samples were collected from each consenting participant for culture and microscopic examination.

### Decontamination and Ziehl-Neelsen (ZN) sputum smear microscopy

Briefly, decontamination was carried out for the sputum samples according to international guidelines using equal volumes with N-acetyl-l-cysteine and sodium hydroxide (NALC-NaOH) (final NaOH concentration 1%) solution. Resuspension of pellets in 1.5 ml of phosphate buffer (pH 6.8) preceded centrifugation [7]. Carbon Fuschin was then used to flood heat-fixed smears. The flooded slide was heated to steam with a flame and let to stand for 10 min, then washed with water and decolorized using 3% acid alcohol. After that, the smear was flooded with malachite green and left to stain for 2 min. This stain was then washed with water and smear air dried and later observed microscopically using X100 oil immersion objective [8]. ZN Staining was carried out for all the 132 patient samples.

### TB cultures

After preparation of sediment to be cultured, a vial of mycobacteria growth indicator tube (MGIT) PANTA (polymyxin B, amphotericin B, nalidixic acid, trimethoprim, azlocillin) containing a lyophilized mixture of antimicrobials was reconstituted with 15.0 ml MGIT growth supplement provided. Using a micropipette, 0.8 ml of the mixture was added to each MGIT tube to be inoculated with specimen including the negative control. Using a sterile pipette, 0.5 ml of the well-mixed processed sample was added to the corresponding labeled MGIT tubes. The tubes were closed tightly and inverted three times to allow proper mixing of the components. The MGIT tubes were then inserted into the BACTEC machine [9] after scanning each tube. The instrument maintains a temperature of 37 °C + or - 1 °C, the optimum growth temperature for *M. tuberculosis*.

MGIT tubes were incubated until the instrument flagged them positive, as for the negative tubes, they were flagged after a maximum of 6 weeks when no growth occurred. The tubes that flagged positive were removed and scanned outside the instrument. This followed visual observation of the tube. Mycobacterial growth tends to appear granular, settles at the bottom of the tube, and are rarely turbid as observed with bacterial or fungal contamination. All 132 samples were cultured.

### Hain genotype MTBDR*plus* and genotype MTBDR*sl*

The Hain assays based on DNA-STRIP technology were performed to enable molecular identification of *M.tb* complex and resistance to RIF and/or INH (MTBDR*plus*) and to FQ, aminoglycosides and ethambutol (MTBDR*sl*). The procedure for both molecular assays was divided into three steps: DNA extraction, multiplex amplification with biotinylated primers and reverse hybridization [8].

### DNA extraction, amplification and hybridization

The bacterial DNA was extracted only from TB positive samples using GenoLyse kit (Hain Lifescience, Nehren, Germany) according to the manufacturer’s instructions [8]. Briefly, 1 ml of the decontaminated sputum was centrifuged for 15 min at 10000×g and supernatant discarded. Then, 100 μl of lysis buffer was added and the sample incubated at 95 °C for 5 min to re-suspend the sediment. After that, 100 μl of neutralization buffer was added, vortexed and centrifuged for 5 min [7]. Multiplex PCR amplification and reverse hybridization of extracted DNA for *rpoB, katG, inhA, gyrA, gyrB, rrs* and *eis* genes was carried out as outlined by HAIN life science [8].

### Evaluation and interpretation of results

The developed strips were pasted on evaluation sheets provided with the kit in the designated fields by aligning the conjugate control and amplification control bands with the respective lines on the sheet. Resistance status was then noted down in the separate columns. Developed mutation bands were consequently related to their respective mutation with the aid of Genolyse package inserts.

### Statistical data analysis

Data were summarized using Minitab 17 software. Continuous data were summarized using mean with standard deviations while categorical variables were summarized using percent (proportion). Graphs were used to summarize the Demographic characteristics of eligible participants and to summarize the prevalence and genetic characterization of mutations in relation to first- and second-line drug resistance.

### Ethical consideration

The samples analyzed in this study were part of STAND (shortening treatment for advancing novel drugs) clinical trial, conducted in collaboration with foreign research institutions that got clearance from the international review board (IRB) as well as the Kenya Medical Research Institute National Ethical Review Committee (KEMRI, ERC).

Written informed consent was obtained from all subjects (or legally acceptable representative) before any procedures were performed.

## Results

### Demographic and clinical characteristics of the subjects

**Fig. 1:**
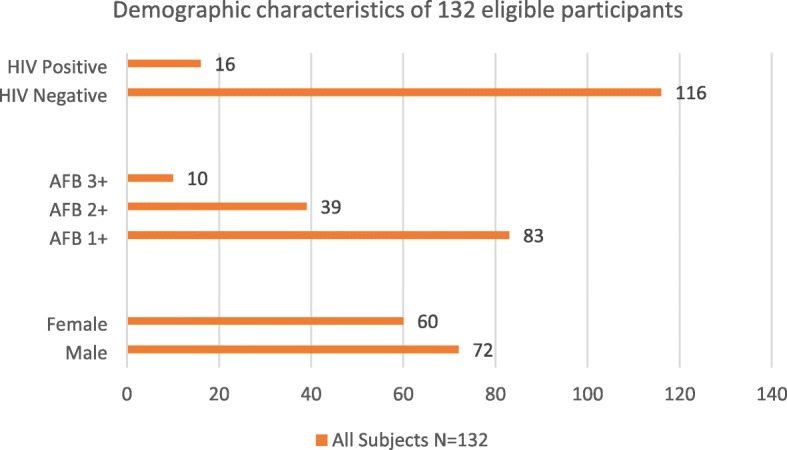
Demographic characteristics of 132 eligible participants

During the period September 2015 to August 2016, a total of 132 smear-positive TB patients were recruited into the study and registered in local TB dispensaries. Their data on HIV status, international union against tuberculosis and lung disease (IUATLD) scaling system for acid-fast bacilli (AFB) smears and gender were recorded (see Fig. 1). These samples (132) were cultured in liquid culture, and Hain assays also conducted, using DNA extracted from the 132 positive liquid cultures. Among them, 72 (54%) patients were male while the remaining 60 (46%) were female. All the patients had a mean age of 26.4 ± 19.4 (SD) and a range of 18 to 60 years. None of the patients were previously treated with first or second-line drugs.

### Genotypic drug resistance profile of the *M. tuberculosis* isolates in the present study

Of the 132 patients that were screened, only two patients showed resistance associated with first- and second-line TB drugs. Of these two patients, one had resistance to INH, while the other depicted a case of MDR resistant to both INH and RIF.

Of the 132 patients screened for the susceptibility to second-line drugs, one cross-resistance was detected for AG/CP antibiotics (kanamycin and amikacin, both AG and capreomycin and viomycin, both CP). On comparing susceptibility between first-line and second-line drug-sensitivity, it was noted that the MDR-TB case had an additional second-line drug resistance while the mono-resistant case had no additional second-line drug resistance.

### Detection of first and second-line drug-resistant mutations in *M. tuberculosis*

To ascertain the molecular basis to INH, RIF, FQ, and the injectable agents, the *rpoB, katG, inhA, gyrA, gyrB, rrs* and *eis* regions were analyzed as per the HAIN assay results and interpreted. Mutations were observed in the S315 T1 of *katG* in 1.5% INH-resistant isolates, with mutations occurring at codon 315. None of the INH-susceptible isolates exhibited mutations. Additionally, one (0.8%) RIF-resistant isolate with mutation S531 L of *rpoB* at codon 530–533 was observed. On the other hand, looking at isolates with resistance to second-line drugs, only one mutation (G1484 T) was observed for *rrs* at nucleic acid position 1484.

**Fig. 2:**
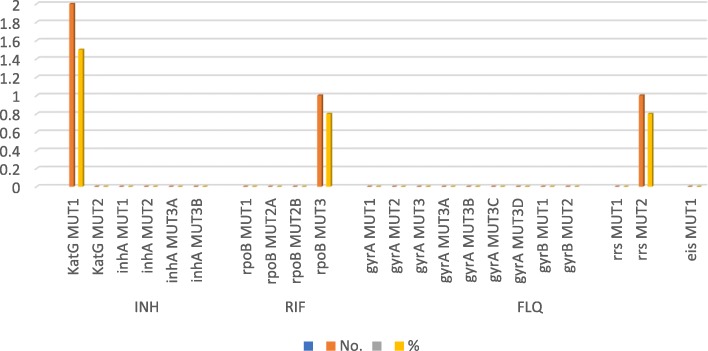
Prevalence and genetic characterization of mutations in relation to first and second line drug resistance

From the 132 isolates examined, no mutation was observed for *inhA*, *gyrA*, *gyrB* and *eis*, relating to low-level INH resistance, FLQ resistance and low-level KAN resistance respectively (see Fig. 2).

## Discussion

This study addressed the prevalence and detection of drug-resistant mutations in *M. tuberculosis* among patients visiting selected health centers in Nairobi, Kenya.

Results in this study put the prevalence rate at 1.5% with 1 (0.8%) of the 132 isolates in the current study, presenting MDR-TB (Table 1). The 132 isolates represented an area in Nairobi that is profoundly affected by MTB; hence analysis of these isolates provides a much exhaustive outlook of both prevalence and detection of drug mutations of these isolates. The prevalence found in this study was much expected considering the patients who were used for the research were drug naïve. Similar results were reported in Tanzania [10] and India [11]. A further explanation into this is that the type of resistance in drug naïve and those who have previously received anti-TB therapy is different. The rate of drug resistance in drug naïve patients (primary resistance) is ordinarily low as compared to acquired resistance (that found in patients who have already received anti-TB therapy). In acquired drug resistance there is the aspect of selective pressure of antibiotic use; hence the Mycobacteria tend to mediate further resistance by use of chromosomal mutations [12]. Higher prevalence rates (23%) were however noted in Ethiopia [13]. The higher prevalence may have been due to the difference in sample size, poor management of TB cases, irregular supply of anti-TB drugs as well as poor treatment compliance.

**Table 1:** Genotypic drug resistance profile of the M. tuberculosis isolates in the present study

	New Cases (*n* = 132)	
Mono drug resistance	No.	%
Isoniazid (INH)	1	0.8
Rifampicin (RIF)	0	0
Fluoroquinolones (FQ)	0	0
Amikacin (AMK)	0	0
Kanamycin (Low resistance)- (KAN)	0	0
Multidrug resistance	No.	%
Isoniazid + Rifampicin	1	0.8
Poly-resistance	No	%
Isoniazid/Rifampicin + Fluoroquinolones/Aminoglycosides	0	0

Delving deep into the data, the isolates presented high-level resistance towards INH, RIF and a cross-resistance was also noted for KAN, AMK, CP and viomycin (VIO). These findings are similar to those previously obtained in Mombasa, Kenya [14] where 8 (3.1%) and 1(0.4%) monoresistance to INH and RIF, were reported respectively. However, no resistance was reported for second-line drugs. This implies that resistance in *M. tuberculosis* isolates is basically ‘multiple’ naturally, the instance it evolves and thus stress the need for continued surveillance and the homogeneity in drug resistance that seems to be occurring in the country [15].

Higher resistance to INH in other countries has also been noted at 5% in Tanzania [16] and 8% in Ethiopia [17]. The subsequently high rates of INH resistance in this study as well as in others may be due to the prolonged use of the drug as prophylactic therapy for TB prevention [18]. This rationale may, however, be disputed considering new research that examines the occurrence of drug resistance due to imperfect drug penetration [19]. When considering imperfect drug penetration, one must develop a perception about drug combinations that is counterintuitive. Take for instance a combination of two drugs with different targets, suppose INH reaches its target while RIF does not. You will find the pathogen evolving resistance to INH and assume that is where the problem is. Instead, it is RIF that isn’t doing its job since it does not reach its intended target and that is the drug that may have to be fixed. However, monoresistance to INH should be well monitored to curb the spread of MDR-TB strains in the area.

It also seems that resistance to the other drugs occurs based on resistance to the first-line drugs of RIF and INH, considering resistance to other drug analogs without involving at least one of these two drugs was exceedingly uncommon (none out of 132 isolates) and that 99.2% of isolates remained susceptible to fluoroquinolones, aminoglycosides and capreomycin. This circumstance is likely associated with the sequential use of distinct anti-tuberculosis drugs for treatment, also, to spread of the MDR strains [20].

In this study, only one case of MDR-TB was detected, accounting for 0.8% of the samples. This agrees with other studies in various countries which encountered the same low levels, countries such as Ethiopia with 1.1% [13] as well as India recording the same percentage [21]. Kidenya et al., also reported prevalence for the past 15 years in Tanzania ranging from 0.4–2.1%, in addition to East Africa at 0.4–4.4% [22]. These findings, however, do not concur with other reports from other countries where the prevalence of MDR-TB was higher such as in Mozambique with 5.8% [23] and Swaziland reporting 7.7% [24]. The divergence may be due to variations in sample size, and population studied, the effectiveness of DOTS strategy and access to health care facilities. A recent study, however, shows that the rise is due to the quality of care in both the private and public sectors, which has fallen short of international standards and desperately need improvement [25].

Various mutations were detected in this study, implying that the scope of mutations that confer resistance to MTB may be much more comprehensive than those proclaimed in the research. In a meager region of amino acids situated between position numbers 507–533 of the *rpoB* gene, a bulk of mutations that confer resistance to RIF (73–100%) are consistently present [26]. The proclaimed epidemiological information implies the RIF resistant strains are extensively disseminated in many regions worldwide. In the current study, the RIF resistant isolate had the mutation S531 L; the most often recorded resistance mutation in various countries [27].

*KatG* gene is the most common region targeted with a bulk of mutations occurring in codon 315 in 30–90% of INH resistant strains. However, this relies on graphical distribution [28]. A significant number of reports imply that resistance of *M. tuberculosis* to INH show mutation at codon 315 [23]. Findings of this study were complementary, displaying mutations at S315 T associated with elevated level of drug resistance to INH. Comparable mutation trends on S315 T have been confirmed at *KatG* gene and have been reported in other studies including Ethiopia [29], Uganda [30]. This indicates possible use of the same drug analogs in the regions. Different drug analogs used in the different areas has an influence on the various drug-resistant mutations observed [31]. Mutations that are observed from the use of the different drug analogs may shed some light on geographical differences accounted for in relation to drug efficacy.

Nevertheless, in this study, gene mutation associated with low-level drug resistance induced by the mutations in the promoter region of *inhA* gene was not detected. Comparable verdicts were reported from research conducted in Ethiopia [29]. As opposed to the current study, low and high-level frequency of *inhA* mutation have been reported in Canada 26% and Tunisia 36.1% [32]. This highlights the importance of surveillance and the heterogeneity in drug resistance that may occur within Kenya.

In the present study, all the gene mutations among strains that are resistant to *rrs* by Genotype MTBDR*sl* assay was detected at nucleic acid position 1484 resulting in the mutation G1484 T. Mutation G1484 T has also been reported to be dominant in various regions [33]. Mutations observed among isolates of *M. tuberculosis* in this study also exhibited cross-resistance to AG/CP and were further resistant to both INH and RIF, suggesting an MDR strain of *M. tuberculosis*. Similar drug resistance trends can be found in other MDR cases in other areas; however, the second-line drug /antibiotic resistance may vary [33]. Mutations related to second line drug resistance in this study were minimal mainly since the patients were drug naïve and had no history of second-line drug treatment. This is unlike cases involving patients who have undergone retreatment failure and had a long history of second-line drug treatment and are more likely to exhibit isolates with mutations linked with resistance to the drugs.

The findings in this study carry some critical implications. First, the high incidence of resistance to INH implies that mutations that are responsible for resistance to INH in this setting are accumulating and an added increase of RIF resistance will eventually lead to MDR-TB. Secondly, the prevalence of MDR-TB in drug naïve patients is still at an all-time low over the years, and this reflects the success of some of the DOTS strategies implemented to manage drug-susceptible TB and prevention measures taken to curb incidences of MDR-TB. Also, considering the rarity of MDR-TB is drug naïve patients, empirical category I regimen can still be used to treat new cases of pulmonary tuberculosis without the risk of treatment failures or exacerbation of drug-resistance. Finally, mutations that are observed from the use of the different drug analogs may shed some light on some of the reported geographical differences in drug efficacy.

In this study, Genotype MTBDR*plus* and MTBDR*sl* assays detect the resistance originating from the *katG*, *rpoB, inhA*, *gyrA*, *gyrB*, *rrs* and *eis* regions as examined. As recommended by the manufacturers’ instructions for usage, a considerable disadvantage of the assay is that few of the mutations that confer resistance to RIF and INH anti-TB drugs are proven. However, the high concordance rate with conventional methods and the rapid time to results make the MTBDR*plus* and MTBDR*sl* assays useful tests for the diagnosis and management of multi-drug resistant tuberculosis.

Additionally, the sample size was limited to ongoing studies and therefore limited in power to detect other mutations within the population as well as the prevalence of MDR-TB in the region. However, our study strength was the fact that the hospitals covered provided a good representation of the region. Though it would be of interest if the study was conducted with a larger sample size.

## Conclusion

This study shows that there is homogeneity when it comes to the type of drug resistance and mutations that occur within the region. The detected prevalence of 1.5% resistance to first- and second-line drug resistant mutations of *M. tuberculosis* in this study calls for intensified drug-resistance surveillance and drug adherence among patients infected with TB.

## Abbreviations

AFB: Acid fast bacilli
AG/CP: Aminoglycosides/Capreomycin
AMK: Amikacin
DNA: Deoxyribonucleic acid
DOTS: Directly Observed Treatment, Short Course
DST: Drug susceptibility Test
FQ: Fluoroquinolone
HIV: Human Immunodeficiency Virus
INH: Isoniazid
IUATLD: International union against tuberculosis and lung disease
KAN: Kanamycin
KEMRI: Kenya Medical Research Institute
MDR: Multi-Drug Resistant
MGIT: Mycobacteria growth indicator tube
NALC: N-acetyl-l-cysteine
NaOH: Sodium hydroxide
NUITM: Nagasaki University Institute of Tropical Medicine
PANTA: Polymyxin B, Amphotericin B, Nalidixic Acid, Trimethoprim, Azlocillin
PCR: Polymerase chain reaction
RIF: Rifampicin
SD: Standard Deviation
TB: Tuberculosis
VIO: Viomycin
WHO: World Health Organization
XDR: Extensively drug-resistant
